# Biocuration - mapping resources and needs

**DOI:** 10.12688/f1000research.25413.2

**Published:** 2020-12-02

**Authors:** Alexandra Holinski, Melissa L. Burke, Sarah L. Morgan, Peter McQuilton, Patricia M. Palagi

**Affiliations:** 1 European Molecular Biology Laboratory, European Bioinformatics Institute (EMBL-EBI), Hinxton, Cambridgeshire, CB10 1SD, UK; 2 Oxford e-Research Centre, Department of Engineering Science, University of Oxford, Oxford, Oxfordshire, OX1 3QG, UK; 3 SIB Swiss Institute of Bioinformatics, Lausanne, 1015, Switzerland

**Keywords:** Biocuration, Career development, Training needs, Biocuration tools, Bioinformatics, Life Sciences

## Abstract

**Background: **Biocuration involves a variety of teams and individuals across the globe. However, they may not self-identify as biocurators, as they may be unaware of biocuration as a career path or because biocuration is only part of their role. The lack of a clear, up-to-date profile of biocuration creates challenges for organisations like ELIXIR, the ISB and GOBLET to systematically support biocurators and for biocurators themselves to develop their own careers. Therefore, the ELIXIR Training Platform launched an Implementation Study in order to i) identify communities of biocurators, ii) map the type of curation work being done, iii) assess biocuration training, and iv) draw a picture of biocuration career development.

**Methods:** To achieve the goals of the study, we carried out a global survey on the nature of biocuration work, the tools and resources that are used, training that has been received and additional training needs. To examine these topics in more detail we ran workshop-based discussions at ISB Biocuration Conference 2019 and the ELIXIR All Hands Meeting 2019. We also had guided conversations with selected people from the EMBL-European Bioinformatics Institute.

**Results:** The study illustrates that biocurators have diverse job titles, are highly skilled, perform a variety of activities and use a wide range of tools and resources. The study emphasises the need for training in programming and coding skills, but also highlights the difficulties curators face in terms of career development and community building.

**Conclusion:** Biocurators themselves, as well as organisations like ELIXIR, GOBLET and ISB must work together towards structural change to overcome these difficulties. In this article we discuss recommendations to ensure that biocuration as a role is visible and valued, thereby helping biocurators to proceed with their career.

## Introduction

Together with bioinformatics, curated databases have become an essential part of modern molecular biology. Databases such as Ensembl ^1^, COSMIC ^2^ and PomBase ^3^ are accessed daily by thousands of people worldwide, illustrating that well-structured life science data, available to all in public repositories, are fundamental to research. Often, these biological databases are annotated by biocurators whose work, in a simplified explanation, is to i) collect scientific data, ii) verify and validate the information collected, iii) add value by structuring it in a logical, consistent and relevant manner and iv) integrate it into databases. Bourne and McEntyre ^4^, in their homage to the profession, considered biocurators as the “museum cataloguers of the Internet age”. We believe biocurators are more than this, they are integral to the modern life sciences and pivotal to good data management. They are the guardians of the integrity and FAIRness of life sciences data.

The art and science of biocuration helps make data Findable, Accessible, Interoperable and Reusable (FAIR) ^5^; it places data into context, makes it more interoperable, adding unique identifiers, licences, and structured descriptions alongside other appropriate metadata. In this respect, biocuration shares some aspects of data stewardship, but there are also some differences. Data stewardship involves improving the FAIRness of a dataset, but doesn’t include adding value to the data, via curation and integration. In many ways, biocuration begins where data stewardship finishes, with data stewards helping researchers to prepare their data for biocurators to add value through integration into appropriate databases.

The European life sciences infrastructure for biological information (ELIXIR; https://elixir-europe.org) is an inter-governmental organisation that brings together bioinformatics resources across Europe and helps researchers to find, analyse, and exchange life science data. In 2016, the ELIXIR Data Platform put in place a process to identify European data resources that are of fundamental importance to research in the life sciences ^6^. Many of these resources are manually curated and ELIXIR recognises the substantial added value that this brings to these resources. Manual biocuration is a highly valued task, and the biocuration efforts of a resource are an important indicator of its quality.

The International Society for Biocuration (ISB; https://www.biocuration.org) counts over 250 members working in over 82 institutions around the globe. The 27th annual Nucleic Acids Research database issue and molecular biology database collection lists 1613 databases, including 59 new databases ^7^. Biocurators not only work in well-known databases but also in *ad-hoc*, often behind the scenes projects where biocuration is needed, be they in academia, non-governmental organisations or private companies. A large, diverse body of individuals and teams are involved in biocuration across ELIXIR. It is anticipated that a number of these may not identify themselves as biocurators *per se* – this could be due to them being unaware of this as a position or career path, or that biocuration is not the main aspect of their job. In this article, we use a broad definition of ‘biocurator’ to encompass anyone who carries out biocuration tasks as part of their work regardless of whether it is the primary focus of their role. 

The ELIXIR Training Platform (TrP), one of the five infrastructure pillars of ELIXIR, aims to strengthen national bioinformatics programmes, grow bioinformatics capacity and competences across ELIXIR member states and empower researchers to use ELIXIR’s services and tools ( https://elixir-europe.org/platforms/training). A large number of biocurators work within the ELIXIR Nodes, and the ELIXIR TrP requires a clear picture of who they are, their profiles, what resources they work on, the tools that they use and, in particular, their training needs. The last survey of biocurators was conducted several years ago in 2011, and in an international context where ELIXIR and the Global Organization for Bioinformatics Learning, Education and Training (GOBLET, https://www.mygoblet.org) did not yet exist. An up-to-date profile of the landscape of biocuration would allow these organisations and employers to take action to recognise and value biocuration, and to plan future training. 

In this research article we describe the outcomes of an ELIXIR Biocuration Implementation Study that aims to i) identify communities of biocurators within ELIXIR and worldwide, ii) review and map the kind of biocuration work being done, for which databases and life science/health domains, iii) assess the capacity requirements for new biocurators and training provision available, and iv) draw a picture of the biocuration career development. We outline a set of recommendations to ensure that the biocuration career path can be even more valued. We urge the community to ensure that the knowledge and skills offered by existing biocurators are shared across ELIXIR Nodes and beyond, and to develop training so it is available to those areas and disciplines where biocurators needs are in highest demand.

## Methods

### Surveys and guided conversations with selected biocurators

In order to map the landscape of biocuration, we first ran a pilot survey with staff at the Wellcome Genome Campus, near Cambridge, UK. The pilot was used to test the survey questions with a smaller audience so that they could be fine-tuned to enable us to undertake the required analysis. This pilot included questions (see *Extended data*: Pilot survey questions ^8^) about the nature of the respondents’ work (job title, location and type of organisation), the tools and resources used in their day-to-day work, training that the respondents have received, and additional training needs. It was sent to all campus staff as we had the specific aim of capturing individuals who may not identify as biocurators but who carry out biocuration-related tasks as a part of their role. The pilot survey was open between 12 December 2018 and 18 January 2019 with reminders sent ten days and one day before the final deadline.

Additionally, we met with four EMBL-European Bioinformatics Institute (EMBL-EBI) biocurators who had replied to the pilot survey to discuss their responses in more detail. The conversations were carried out in person by Alexandra Holinski and Melissa Burke at EMBL-EBI and took the form of a guided conversation (see *Extended data*: Questions to guide conversations with biocurators ^8^) with answers captured and transcribed directly, rather than recording and transcribing later.

The final survey (see *Extended data*: Global survey questions ^8^) was a revised version of the pilot. Revisions were made where questions led to overly general and broad answers and to introduce relevant topics raised in the conversations. This survey was disseminated globally by making use of Wellcome Genome Campus, ELIXIR, International Society for Computational Biology (ISCB), ISB, the SIB Swiss Institute of Bioinformatics and GOBLET mailing lists. It was also publicised via Twitter and LinkedIn with reminders and retweets sent at regular intervals. To reach biocurators in industry, the survey was promoted at the EMBL-EBI Industry Programme quarterly meeting and was disseminated through their mailing list. This global survey was open between 4 March 2019 and 1 May 2019. Both the pilot and global survey were conducted online using SurveyMonkey ( https://www.surveymonkey.co.uk).

### Workshops at ISB Biocuration Conference 2019 and ELIXIR All Hands Meeting 2019

Two workshops were organised to present and discuss the results of the global survey. The workshops were designed to actively capture the participants’ feedback on the survey results and their thoughts on several key questions. In both workshops attendees were asked to note down their answers to the questions on post-it notes that were collected on whiteboards. The slides presented in the workshops are publicly available ^9– 12^. They include photographs and transcripts of the post-it notes collected during group discussions.

The first workshop was held on 7 April at the 11th ISB Biocuration Conference 2019 in Cambridge, UK. Twenty-eight people attended the workshop where we presented the interim survey results collected between 4 and 22 March 2019. In this workshop we directed the following questions to the participants: i) How did you get into the field? Have you left? What do you do now? ii) How long did it take you to feel “fully” trained as a biocurator? iii) What skill/piece of knowledge was the most difficult to learn when becoming a biocurator? iv) How would you encourage others into the field of biocuration? v) How can we engage with others whose role is partially biocuration; or those sitting outside of traditional academic/research centres? vi) What career support would you like to see for those in biocuration; where would you like your skills to take you?

The second workshop was conducted on 19 June 2019 at the ELIXIR All Hands Meeting in Lisbon, Portugal. Twenty-one people attended the workshop in which we presented the full results of the survey. The participants were asked to comment on the following questions: i) How can ELIXIR support biocurators? ii) What are your experiences with/thoughts on community biocuration? iii) Anything else you would like to tell us?

### Analysis and interpretation

In our analysis, we focus on the outcomes of the global survey, the conversations with EMBL-EBI biocurators and the two workshops. Analysis of all data was performed by A.H. and M.B. Where free text answers were provided in the global survey, themes across the answer sets were initially independently identified and categorised by A.H. and M.B. These individual sets were then combined to form the final answer sets provided below. For example, for the question ‘Please provide a short description of your current work.’ the answers were sorted into categories based on the tasks mentioned. A.H and M.B took a similar approach for analysing the guided conversations. Themes across the answers were independently identified and categorised and the individual sets were combined. For the word clouds, responses that make no sense were removed from the word lists, e.g. fff or wsx. The responses were edited for case, e.g. Bionformatician vs. bioinformatician, to ensure that each term is only displayed once in the word cloud. Word clouds were created with WordClouds.com ( https://www.wordclouds.com/). It is worth noting that the outcomes of the pilot survey do not differ greatly from the results of the global survey.

### Ethical considerations, data availability and GDPR statement

Our study was designed to collect data on biocuration practices and training needs, with only a limited set of sensitive personal data (email and name) which were not to be used as part of the reporting set. Given the nature of our study, we therefore deemed it unnecessary to undergo formal ethical review via an ethics committee. For participants, all details relating to the involvement in the study, including how the information they provided would be used and where results would be reported, was provided in both an invitation email and at the start of the survey. Participant consent was presumed based on their completion of the survey.

This study was conducted in accordance with GDPR. Directly identifiable data (e.g. name, email address) was not collected from all respondents as this was an optional element of the survey. The nature of a number of the responses provided does however mean that it may be possible to identify individuals in specific positions due to their unique job titles and affiliated institutions. Given this potential for identification we are not providing raw data outputs from the survey and transcripts from the guided conversations.

## Results

### Communities of biocurators within ELIXIR and worldwide

**Figure 1.**
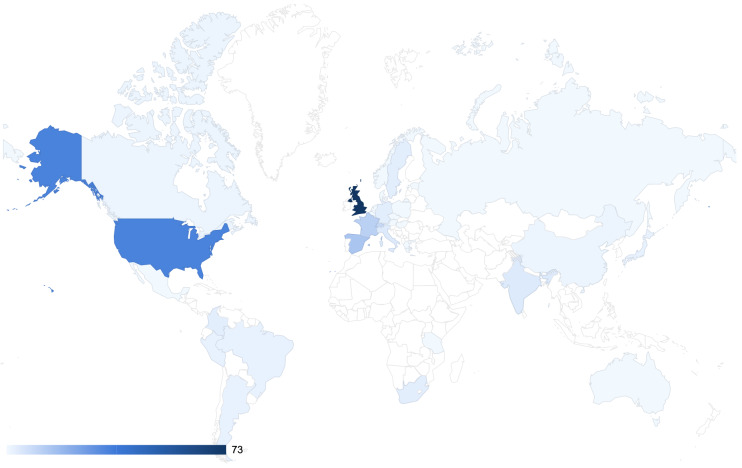
Countries in which biocurators work. The map represents the countries in which those biocurators who responded to the survey work. The colour shading in the map indicates the number of respondents from each country.

The global survey received 212 responses with most from Europe and the USA ( Figure 1).

**Figure 2.**
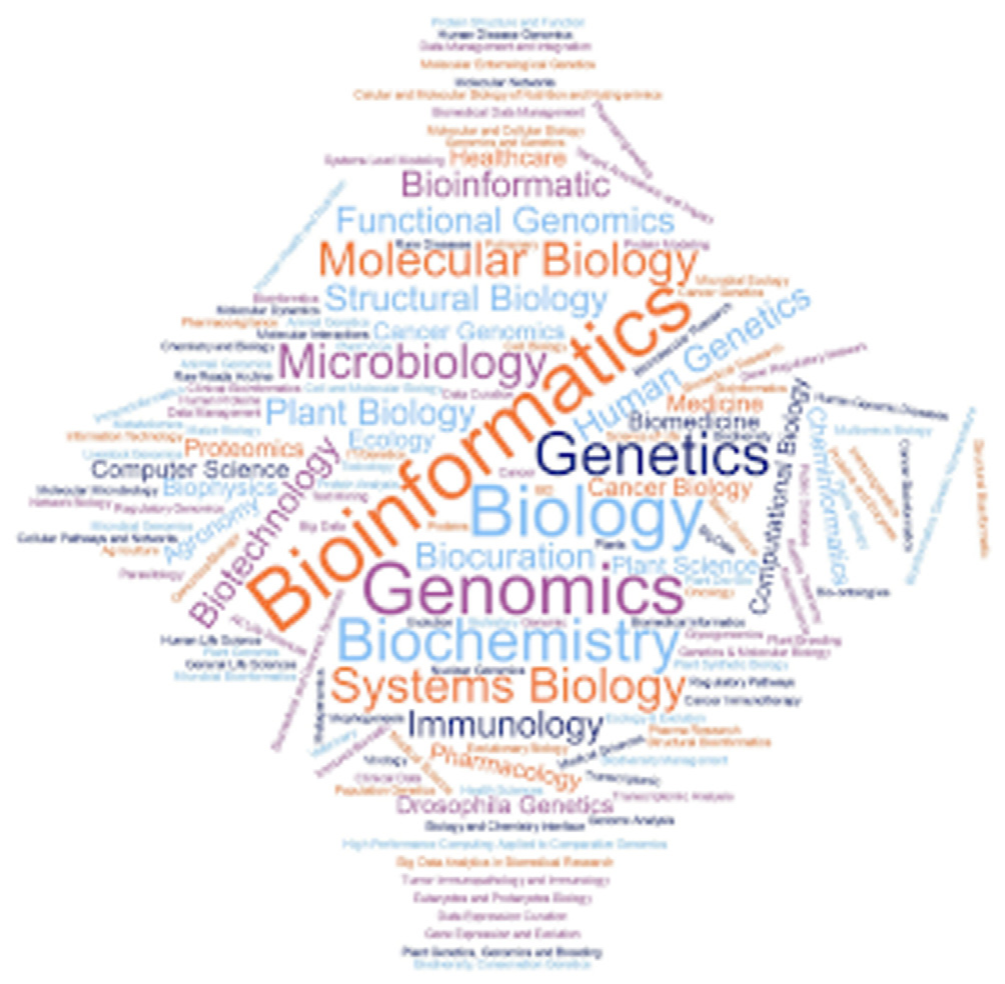
The variety of life science domains that biocurators belong to. The size of the text in the word cloud is related to how often particular life science domains were mentioned by respondents. The colours were chosen to create a better contrast between the words.

Respondents work in 91 different organisations, with 84% of respondents belonging to academic institutions. Eight percent of the respondents work in an industrial organisation and 1% span both academia and industry (see *Underlying data:* Global_survey_deidentified ^8^). Among the named academic institutes, 25% of respondents belong to international biocuration hubs, including EMBL-EBI, the Wellcome Sanger Institute and SIB. In free text answers, respondents indicated that they work across a wide range of life science domains, with the most commonly mentioned being bioinformatics, genomics, genetics, biology and biochemistry ( Figure 2).

**Figure 3.**
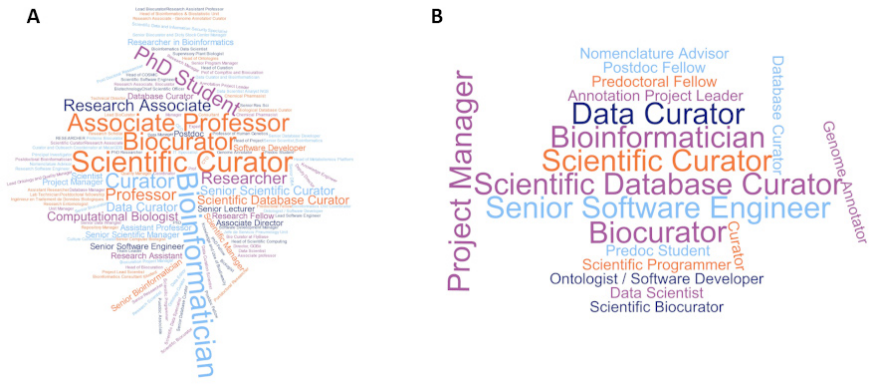
Job titles provided by respondents of the global survey. The figure shows all responses ( **A**) and responses filtered by respondents from EMBL-EBI ( **B**). The size of the text in the word cloud is related to how often particular job titles were mentioned by respondents. The colours were chosen to create a better contrast between the words.

The survey also shows that the job titles of respondents are diverse, including uncommon titles, such as knowledge engineer, data editor, repository manager, and data wrangler (the diversity of job titles is reflected in the word cloud Figure 3A). Even for biocuration hubs, such as the EMBL-EBI, a large variety of job titles was reported ( Figure 3B).

This diversity of job titles makes biocurator positions difficult to identify, and this may impede the building of a sustainable community. In both the guided conversations and workshops, biocurators emphasised that the inconsistency of job titles hampers their career planning. Biocurators may not easily find relevant job adverts or in fact identify with them based on the title alone, and employers may have difficulty in determining what skills and knowledge to associate with which job title.

### The type of biocuration work being done

The survey shows that biocurators perform a diverse range of activities and that biocuration tasks are undertaken in a variety of roles. From a list of biocuration activities, the tasks most often selected in the survey were data annotation (69 of 166 respondents - 42%), ontology and controlled vocabulary application (62 of 166 respondents - 37%), data analysis (48 of 166 respondents - 29%), and literature curation (47 of 166 respondents - 28%) (see *Underlying data:* Global_survey_deidentified ^8^). In addition, many respondents indicated in free text that they also take part in database, pipeline and method development, web-interface design, help-desk and user requests, teaching, outreach, team management, grant writing, and ontology development (see *Underlying data:* Global_survey_deidentified ^8^). This variety of tasks makes it difficult to define a clear biocuration profile.

Participant responses to the survey, our conversations with biocurators and the workshops also make it clear that the precise nature of biocuration methods employed depends on the area in which they work. This is reflected in the 400 different tools listed in the survey (see *Underlying data:* Tools and resources ^8^) as being used in biocuration activities. These resources can be broadly clustered into public databases and tools, literature search tools, ontology services, scripting languages and in-house biocuration tools; many of them are specific to the biocurators’ roles and particular life science domains in which they work. Biocurators that we spoke to and workshop participants emphasised that this makes common biocuration pipelines difficult to identify and implement, and complicates efficient knowledge exchange and community building amongst biocurators.

### Biocuration training

**Figure 4.**
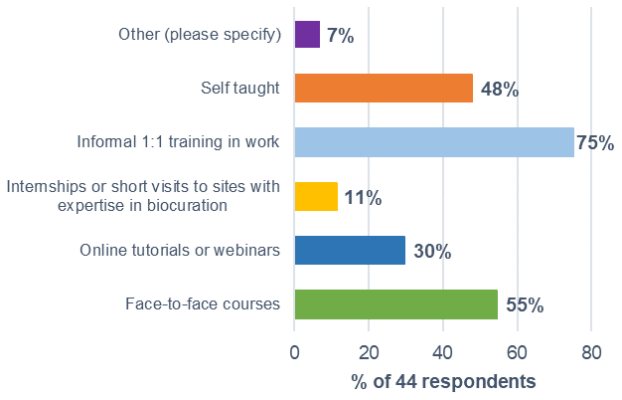
The type of training survey respondents had access to. Respondents could choose several answers. This question was answered by 44 respondents. The percentage of respondents who chose the respective training type is also indicated in the figure.

In our conversations with biocurators and at the workshops, biocurators emphasised that it can take a long time to be trained as a biocurator and most agreed that biocuration is a continuous learning process. According to the survey, 46 of 164 (28%) respondents said that they have received training in biocuration, while the rest said that they have not received any training (see *Underlying data:* Global_survey_deidentified ^8^). Training was mainly received in the form of one-to-one teaching and self-directed training, or via face-to-face courses ( Figure 4). Respondents view one-to-one and self-directed training as the most impactful forms. The in-house nature of this training combined with the specific nature of biocuration tasks suggests that biocuration knowledge gained in one role is not necessarily easily transferable to another.

From the responses provided, we collated a list of face-to-face and online training courses, materials and providers (see *Underlying data:* Biocuration training course list ^8^), which may be relevant to biocurators. This course collection is not exhaustive and may again reflect the nature of the informal 1:1 and in-house training many respondents said they had received.

In the survey, from free-text answers, the most commonly indicated training needs are programming/scripting/coding (mentioned in 18 of 134 responses - 13%), development and use of ontologies (mentioned in 16 of 134 responses - 12%), and database management (mentioned in 10 of 134 responses - 7%) (see *Underlying data:* Global_survey_deidentified ^8^). Survey respondents and biocurators that we spoke to highlighted that computational skills, such as programming/scripting/coding and extracting relevant data from the literature, are decisive for a successful career development yet are the most difficult to learn. Other skills that were highlighted as being essential for a successful biocuration career include biological knowledge (mentioned in 41 of 124 responses - 33%), attention to detail (mentioned in 23 of 124 responses - 19%), patience (mentioned in 11 of 124 - 9%), communication (mentioned in 8 of 124 responses - 6%) and curiosity (mentioned in 8 of 124 responses - 6%) (see *Underlying data:* Global_survey_deidentified ^8^). These views were reiterated and emphasised by biocurators that we spoke to individually and at the workshops.

### Career development

Our conversations with biocurators and the workshop discussions make it clear that biocurators typically enjoy their jobs. It is an attractive and rewarding career for skilled individuals who like data analysis, scientific writing and communication. Biocurators who participated in our study stated that the job offers the opportunity to move away from the lab but still work in a research environment without experiencing the pressure of publishing papers and offers a good work-life balance. The outcomes from the conversations and workshops, however, also suggest that biocuration is an invisible career; many people within the scientific community are unaware of biocuration as a career and undervalue the impact of biocuration. All four EMBL-EBI biocurators that we spoke to as well as some workshop participants commented that they had not previously heard of the role before becoming a biocurator. Some also felt that biocuration is underappreciated, which is reflected in difficulties obtaining funding for biocuration work.

In summary, our findings show that biocurators are a highly skilled group of individuals, however a lack of sustainable community building contributes to the fact that new biocurators are difficult to recruit, and that existing biocurators are unaware of biocuration training and future career opportunities and may feel isolated in their job.

## Discussion and recommendations

Modern science, whatever the discipline, is turning into something of a data science. This seems particularly true of the life sciences, where the explosion in omics data, followed by an increase in publications and linked datasets, has led to a massive overall increase in freely available structured and unstructured biological data. Biocuration, the action of combining data from multiple sources, and adding value by converting them into a coherent, consistent and structured interoperable dataset, has never been more essential to enable the understanding, digestion and interpretation of these data.

In this ELIXIR Implementation Study, we asked biocurators from across the world for their thoughts on the state of biocuration, both in terms of the day-to-day technicalities - what tools they use, what skills they need - and the more long range subjects of training and career opportunities. We acknowledge that the survey may not have reached all biocuration communities due to, for example, the preference of certain social media channels in different countries or the lack of awareness of ISB and ELIXIR in certain (geographical) communities. Nevertheless, we believe that the results are a fair representation of the varied views and experiences of biocurators worldwide. Our study highlights the diversity of roles and experience within the biocuration community but also reinforces the need for improved recognition of this work and career.

The day-to-day activities of biocurators are amazingly diverse and include both biocuration and non-biocuration tasks such as training, outreach, website design and project management. As noted by Samili and Vita ^13^, this highlights that biocurators possess many transferable skills in addition to their scientific and biocuration expertise, characteristics that are highly valued by employers. Our survey shows that biocurators in different resources work quite differently, using different tools and following different workflows and pipelines. There are some efforts to encourage biocurators in different resources to streamline their work by following the same biocuration workflows ^14^. Steps have been taken to encourage this streamlining through the addition of biocuration-related databases, curation tools and standard metadata records to FAIRsharing ^9, 11^ (an ELIXIR Recommended Interoperability Resource and the ELIXIR Registry of Standards). However, it is clear from our survey that the diversity of biocuration practices and the data curated mean that a universal biocuration tool or workflow are still some time away.

Having said that, some commonalities in the experience of biocurators can be found. For instance, the clear need for more training on programmatic or coding skills, such as Python, and data modelling skills such as the creation, maintenance and use of ontologies. This is in line with a 2011 survey of biocurators carried out by the ISB in which 55% of respondents thought that better training in computer languages would be beneficial for their jobs, and 43% indicated that they would benefit from better training in bioinformatics ^15^. Similarly, in response to a 2017 survey of biocuration training needs conducted during the development of the Postgraduate certificate in Biocuration at the University of Cambridge, 32% of respondents rated ‘Application of programming to curation tasks - e.g. scripting in Python as their top training need (S.L. Morgan, EMBL European Bioinformatics Institute; personal communication). From our discussions, it became clear to us that it is essential for biocurators to learn coding skills, not only to be able to wrangle data and perform tasks more efficiently but to facilitate discussion with database developers and gain a greater understanding of how the data they curate is represented in a database.

In our survey, 28% of respondents indicated that they had received biocuration training and some training courses were specifically named by respondents. It is possible that this relatively low figure is due to the way in which respondents interpreted the question. For example, respondents might not necessarily equate “training in a programming language”, “1:1 training” or “self-directed training” as “biocuration training”. However, a low level of formal training was also reported in response to the survey of biocuration training needs conducted during the development of the Postgraduate certificate in Biocuration at the University of Cambridge. In that survey, 12% of respondents had received training in the form of formal short courses while 88% had received informal 1:1 training in work (S.L Morgan, personal communication). The specific nature of biocuration tasks observed in our survey calls for a shift in the way that we teach biocuration skills. There is a need to develop bioinformatics training that is targeted to biocurators (e.g. programming skills for biocuration related tasks vs programming for research related tasks). Ensuring that all biocurators are up to speed with the latest biocuration and data stewardship best practices should increase the standard and consistency of biocuration while providing transferable skills that improve career mobility.

The lower reported level of formal training suggests that a potential challenge for biocurators is in being able to find available and/or appropriate training courses. An absence of training courses will also affect community building, with fewer opportunities for biocurators to meet, network and share expertise. To facilitate expanded training for biocurators, training providers should make their courses and training materials easier to find, for example by sharing them in portals such as ELIXIR’s TeSS (the ELIXIR Training and Events Portal; https://tess.elixir-europe.org/) ^12^, and by adopting FAIR principles to ensure that they are appropriately tagged and described ^16^. This would allow democratisation of training efforts such that they expand out from centres of excellence (such as the EMBL-EBI or SIB) to independent biocurators. Some training events and materials listed in the survey have already been indexed in TeSS ^12^, which will allow the identification of gaps in training provision.

Our study also highlights the need for improved career support. This sentiment seems to have changed little from the 2011 ISB survey, in which 82% of respondents felt concerned about future work opportunities ^15^. It can take many years of training and experience to become a biocurator, yet currently, salaries and more importantly job security, often do not reflect this investment. Key barriers to career progression highlighted in our survey are the diversity of job titles, which can make relevant opportunities difficult to find (and diverse in their nature), alongside a lack of recognition of the skills and the role of biocurators both by those performing biocuration activities and in the wider scientific community.

Biocurators that participated in the workshop discussions indicated that one way to address these challenges is to establish stronger links between academia and industry to create a wider and more diverse biocuration community, facilitate knowledge exchange and enable career planning. Another way biocurators can be helped is through appropriate credit, both in terms of citation of the resources they maintain and personal credit through nanopublications, annotation credits and other attributions displayed and linked via their OrCID ^17^.

Ultimately, we perhaps need a new model of careers in biocuration (especially for those individuals for whom biocuration is their primary role), such as those being developed for Research Software Engineers ^18, 19^ and Data Stewards ^20^. In these settings, centralised teams of research software engineers or data stewards have been created, and this has led to improved knowledge exchange, peer support and career sustainability ^19, 20^. For example, within a University environment, a pool of dedicated biocurators could be created, ready to be deployed where need and experience allows, and supported with local funding, so the biocurators themselves have job security.

We urge funding agencies to recognise the importance of biocuration and to fund both databases and biocuration training activities appropriately. In addition, we recommend that both academia and industry provide clearer career structures for scientists whom biocuration is their primary activity, given their role and impact will only grow. We call on everyone, from the ISB, ELIXIR, academia, industry, and biocurators themselves, to work together to create a more integrated global community of biocurators, to share expertise, training initiatives and best practices.

## Conclusion

Biocuration activities have a vital role in the life science data ecosystem, but are often undervalued. These skills, both innate and taught, are highly prized and fundamental not only to the preservation and FAIRness of life science data but are also transferable to other data science and knowledge management roles. The issue we face is one of education. Sometimes scientists performing biocuration activities do not realise the value and rarity of their skills and in many cases researchers and managers do not realise or appreciate the work that biocurators perform. To future-proof this career and the valuable work that biocurators do, organisations, employers and biocurators themselves must act as champions for biocuration and advocate for structural changes. We believe organisations like ELIXIR, ISB and GOBLET have the mandate to actively support the biocurators in their scientific endeavours, work towards raising awareness about the impact that biocuration has within different scientific communities and to convince team leaders of the importance and value of skilled biocuration work. Organisations can also act to facilitate, encourage and support knowledge exchange between biocuration communities. Biocurators themselves have a role to play in championing the work that they do, engaging with the researchers who rely on their expertise and in training the future generation of biocurators. By doing this, organisations and individuals can contribute to the creation of a sustainable and visible biocuration community. We hope the work we have presented here continues this educative process as we continue to define the profession of biocuration.

## Data availability

### Underlying data

The nature of a number of the responses provided means that it may be possible to identify individuals in specific positions due to their unique job titles and affiliated institutions. Given this potential for identification we are not providing raw data outputs from the survey. Instead we provide the de-identified data on Zenodo from the global survey.

The nature of the responses provided in the guided conversations and the fact that all four participants are staff of EMBL-EBI mean that it may be possible to identify the individuals. Given this potential for identification, we do not provide the transcripts but a themed summary of the responses. Information that may lead to the identification of individuals has been redacted.

Data relating to the workshops can be found in the slide sets from these workshops: https://doi.org/10.7490/f1000research.1116798.1 ^9^ and https://doi.org/10.7490/f1000research.1117413.1 ^10^.

Zenodo: Biocuration - mapping resources and needs - Underlying data, http://doi.org/10.5281/zenodo.3991737 ^8^.

This project contains the following underlying data:

- Global_survey_deidentified.xlsx (de-identified responses to the global survey)
  ○ This file includes de-identified responses to the survey questions. Responses that may lead to the identification of respondents have been redacted. Free text responses to questions 6, 14 and 15 have been categorised into tasks, topics and skills, respectively.
- Bar graphs of global survey.xlsx (quantitative responses to multiple choice questions in the global survey. For some questions, respondents could choose more than one option)
- Tools and resources.xlsx (Tools and resources used for biocuration work and listed by the respondents of the global survey)
- Biocuration training course list.xlsx (formal training courses listed by respondents of the global survey)
- Themed summary of the responses given in the guided conversations - deidentified.docx

### Extended data

Zenodo: Biocuration - mapping resources and needs - *Underlying data,* http://doi.org/10.5281/zenodo.3991737 ^8^.

This project contains the following underlying data:

- Pilot survey questions.docx (questionnaire sent to staff of Wellcome Genome Campus)
- Questions to guide conversations with biocurators.docx (conversation guide outlines the type of questions to be asked)
- Global survey questions.docx (globally disseminated questionnaire revised on the basis of the pilot survey)

Data are available under the terms of the Creative Commons Attribution 4.0 International (CC-BY 4.0).
