# Supplementary material for: Biocuration - mapping resources and needs
Source: F1000Res. 2020 Dec 2;9:ELIXIR-1094. Originally published 2020 Sep 4. [Version 2] doi: 10.12688/f1000research.25413.2 (PMC7590901; doi:10.12688/f1000research.25413.2)
Supplement: Peer review history

# Peer review: Reviewer response for version 2

**DOI:** 10.5256/f1000research.30989.r75628

**Published:** 2020-12-02

**Authors:** Luana Licata

**Affiliations:** 1 Bioinformatics and Computational Biology Unit, Department of Biology, University of Rome 'Tor Vergata', Rome, Italy

The revised version of the article is more comprehensive and useful to clarify the rationale behind the study.

Is the work clearly and accurately presented and does it cite the current literature?

Yes

If applicable, is the statistical analysis and its interpretation appropriate?

Not applicable

Are all the source data underlying the results available to ensure full reproducibility?

Partly

Is the study design appropriate and is the work technically sound?

Partly

Are the conclusions drawn adequately supported by the results?

Yes

Are sufficient details of methods and analysis provided to allow replication by others?

Yes

Reviewer Expertise:

Biocuration, Molecular and Causal interactions, Community Standard Formats

I confirm that I have read this submission and believe that I have an appropriate level of expertise to confirm that it is of an acceptable scientific standard.

# Peer review: Reviewer response for version 1

**DOI:** 10.5256/f1000research.28042.r72814

**Published:** 2020-10-26

**Authors:** Luana Licata

**Affiliations:** 1 Bioinformatics and Computational Biology Unit, Department of Biology, University of Rome 'Tor Vergata', Rome, Italy

This work presents the result of an implementation study on biocuration needs.

 The article describes the results coming from the analysis of an online SurveyMonkey polls, filled out by over 200 curators from 33 countries around the globe and integrated with the results of few interviews with a subset of curators and with the outcomes of two questionnaires performed during two workshops.

 The implementation study aimed to define four points;

1. Identify communities of biocurators
2. Map the type of curation work being done
3. Assess biocuration training
4. Draw a picture of biocuration career development.

The global survey was useful to identify biocuration communities, the types of curation work and to understand the training needs while the interviews and workshops questionnaires were useful to outline career development and communities need.

 In the paper, the study is well explained and the conclusion well described.

 However, I think the study design miss some aspects that would have make the result more comprehensive and consistent.

 While the global survey analysis has been performed on a wide and geographically well distributed biocuration community, the interviews analysis not.

 In my opinion, the guided conversations and the selection of biocurators would have benefited from the involvement of a wider number of curators, such as for example, curators coming from different academia and research institutes where the figure of biocurator is less recognised and supported compared to the EMBL-EBI. The interview of a wider number of curators, it could have highlighted and arisen further needs and pain points.

 Moreover, the paper described two questionnaires performed during two workshops held during the 11th ISB Biocuration Conference 2019 and the ELIXIR All Hands Meeting 2019 but the results of these surveys are not explained in full detail and are not available in the supplementary data. There is only a link to the slides presented in the workshops. It would be very useful a summary table or a figure on the results of the two workshops.

 Nevertheless, I think that the conclusions arising from this study are well drawn and pointed out the main needs of the biocuration community.

 I particularly liked the final recommendation and suggestions. I believe that this article will help to increase the appreciation and understanding of biocuration work by scientific communities and the support by Academia and Research Institutes.

Is the work clearly and accurately presented and does it cite the current literature?

Yes

If applicable, is the statistical analysis and its interpretation appropriate?

Not applicable

Are all the source data underlying the results available to ensure full reproducibility?

Partly

Is the study design appropriate and is the work technically sound?

Partly

Are the conclusions drawn adequately supported by the results?

Yes

Are sufficient details of methods and analysis provided to allow replication by others?

Yes

Reviewer Expertise:

Biocuration, Molecular and Causal interactions, Community Standard Formats

I confirm that I have read this submission and believe that I have an appropriate level of expertise to confirm that it is of an acceptable scientific standard.

# Response

**Published:** 2020-11-24

**Authors:** Alexandra Holinski

Dear Luana Licata,

 Thank you for taking the time to review our article and for your valuable feedback on our project. We would like to address two of your comments:

 1)  “While the global survey analysis has been performed on a wide and geographically well distributed biocuration community, the interviews analysis not. In my opinion, the guided conversations and the selection of biocurators would have benefited from the involvement of a wider number of curators, such as for example, curators coming from different academia and research institutes where the figure of biocurator is less recognised and supported compared to the EMBL-EBI. The interview of a wider number of curators, it could have highlighted and arisen further needs and pain points.”

 The guided conversations served to explore the questions asked in the pilot survey in more detail and to identify additional relevant topics for inclusion in the global survey. We agree that one-to-one interviews with a wider number of curators would have been useful, however due to time constraints we were unable to do so. To efficiently gather more diverse and detailed insight into the biocuration landscape we made use of group discussions during the workshops held at ISB 2019 and the ELIXIR all hands 2019 meetings as described in the manuscript. These workshops included a global audience of biocurators. By using this approach we were able to gather a broader set of ideas through the workshops than would have been possible through one-to-one interviews.

 2)  “Moreover, the paper described two questionnaires performed during two workshops held during the 11th ISB Biocuration Conference 2019 and the ELIXIR All Hands Meeting 2019 but the results of these surveys are not explained in full detail and are not available in the supplementary data.”

 In both workshops we did not perform questionnaires but held group discussions in which we asked attendees to note down their answers to specific questions on post-it notes which we collected on whiteboards. This is outlined in the “Methods” (paragraph: Workshops at ISB Biocuration Conference 2019 and ELIXIR All Hands Meeting 2019). Photographs and transcripts of the post-it notes are included in the slides from the workshops (referenced in the article). We understand that this is not clearly specified in the “Methods”, and have revised this section accordingly.

 We hope that our responses help to clarify the rationale behind the study and welcome any further comments.

 Kind regards,

 The authors

# Peer review: Reviewer response for version 1

**DOI:** 10.5256/f1000research.28042.r72815

**Published:** 2020-10-20

**Authors:** Tanya Berardini

**Affiliations:** 1 The Arabidopsis Information Resource, Phoenix Bioinformatics, Fremont, CA, USA

The manuscript details the analysis of a global survey of biocuration work using the results of several in person interviews, online SurveyMonkey polls, and a couple of workshops to identify biocurators and the types of work that they perform.  It is apparent that the community is diverse not just in training, background,and job titles, but also in the skill sets and tools that are employed in day to day tasks.

 I appreciate the details provided by the study and the emphasis on the need for more broader scientific engagement to understand and appreciate this type of work.

 I recommend acceptance without revisions and hope that the indexing and broad dissemination of this paper will result in a greater appreciation for and support of the biocuration community.

Is the work clearly and accurately presented and does it cite the current literature?

Yes

If applicable, is the statistical analysis and its interpretation appropriate?

Not applicable

Are all the source data underlying the results available to ensure full reproducibility?

Yes

Is the study design appropriate and is the work technically sound?

Yes

Are the conclusions drawn adequately supported by the results?

Yes

Are sufficient details of methods and analysis provided to allow replication by others?

Yes

Reviewer Expertise:

biocuration, community engagement, data resource sustainability

I confirm that I have read this submission and believe that I have an appropriate level of expertise to confirm that it is of an acceptable scientific standard.
